# Plasma cell neoplasms and related entities—evolution in diagnosis and classification

**DOI:** 10.1007/s00428-022-03431-3

**Published:** 2022-11-21

**Authors:** Falko Fend, Ahmet Dogan, James R. Cook

**Affiliations:** 1 Institute of Pathology and Neuropathology and Comprehensive Cancer Center, Tübingen University Hospital, Tübingen, Germany; 2 Memorial Sloan Kettering Cancer Center, New York, NY 10065 USA; 3 Department of Clinical Pathology, Cleveland Clinic, Cleveland, OH 44195 USA

**Keywords:** Plasma cell neoplasms, Monoclonal gammopathy of undetermined significance, Multiple myeloma, Lymphoplasmacytic lymphoma

## Abstract

**Abstract:**

Plasma cell neoplasms including multiple myeloma (MM) and related terminally differentiated B-cell neoplasms are characterized by secretion of monoclonal immunoglobulin and stepwise development from a preneoplastic clonal B and/or plasma cell proliferation called monoclonal gammopathy of undetermined significance (MGUS). Diagnosis of these disorders requires integration of clinical, laboratory, and morphological features. While their classification mostly remains unchanged compared to the revised 2016 WHO classification and the 2014 International Myeloma Working Group consensus, some changes in criteria and terminology were proposed in the 2022 International Consensus Classification (ICC) of mature lymphoid neoplasms. MGUS of IgM type is now divided into IgM MGUS of plasma cell type, precursor to the rare IgM MM and characterized by MM-type cytogenetics, lack of clonal B-cells and absence of *MYD88* mutation, and IgM MGUS, NOS including the remaining cases. Primary cold agglutinin disease is recognized as a new entity. MM is now formally subdivided into cytogenetic groups, recognizing the importance of genetics for clinical features and prognosis. MM with recurrent genetic abnormalities includes MM with *CCND* family translocations, MM with *MAF* family translocations, MM with *NSD2* translocation, and MM with hyperdiploidy, with the remaining cases classified as MM, NOS. For diagnosis of localized plasma cell tumors, solitary plasmacytoma of bone, and primary extraosseous plasmacytoma, the importance of excluding minimal bone marrow infiltration by flow cytometry is emphasized. Primary systemic amyloidosis is renamed immunoglobulin light chain amyloidosis (AL), and a localized AL amyloidosis is recognized as a distinct entity. This review summarizes the updates on plasma cell neoplasms and related entities proposed in the 2022 ICC.

**Key points:**

*• Lymphoplasmacytic lymphoma can be diagnosed with lymphoplasmacytic aggregates in trephine biopsies < 10% of cellularity and evidence of clonal B-cells and plasma cells*.

*• IgM MGUS is subdivided into a plasma cell type and a not otherwise specified (NOS) type.*

*• Primary cold agglutinin disease is recognized as a new entity*.

*• The term “multiple myeloma” replaces the term “plasma cell myeloma” used in the 2016 WHO classification*.

*• Multiple myeloma is subdivided into 4 mutually exclusive cytogenetic groups and MM NOS*.

*• Minimal bone marrow infiltration detected by flow cytometry is of major prognostic importance for solitary plasmacytoma of bone and to a lesser extent for primary extraosseous plasmacytoma*.

*• Localized IG light chain amyloidosis is recognized as a separate entity, distinct from systemic immunoglobulin light chain (AL) amyloidosis*.

## Introduction

Plasma cell neoplasms and related entities are derived from terminally differentiated B-cells and characterized by secretion of a monoclonal immunoglobulin in most cases. These disorders share their stepwise evolution from the preneoplastic precursor lesion termed monoclonal gammopathy of unknown significance (MGUS) and the common presence of symptoms and complications related to excess quantities of clonal immunoglobulins or their abnormal deposition in tissues. Given the specific disease features due to the abnormal clonotypic immunoglobulin, which can be detected as a serum biomarker at the preclinical level, the classification of these neoplasms requires the integration of clinical and laboratory features for staging and therapy decisions. The changes proposed in the 2022 International Consensus Classification of Mature Lymphoid Neoplasms are mostly minor and relate both to issues of terminology and to more precise disease definitions and refined diagnostic criteria. The recognition of the major importance of primary genetic alterations resulted in the formal subdivision of multiple myeloma into cytogenetic groups [13]. This article briefly summarizes the current diagnostic criteria for these disorders and describes the updates proposed in the 2022 ICC.

## IgM MGUS

**Fig. 1:**
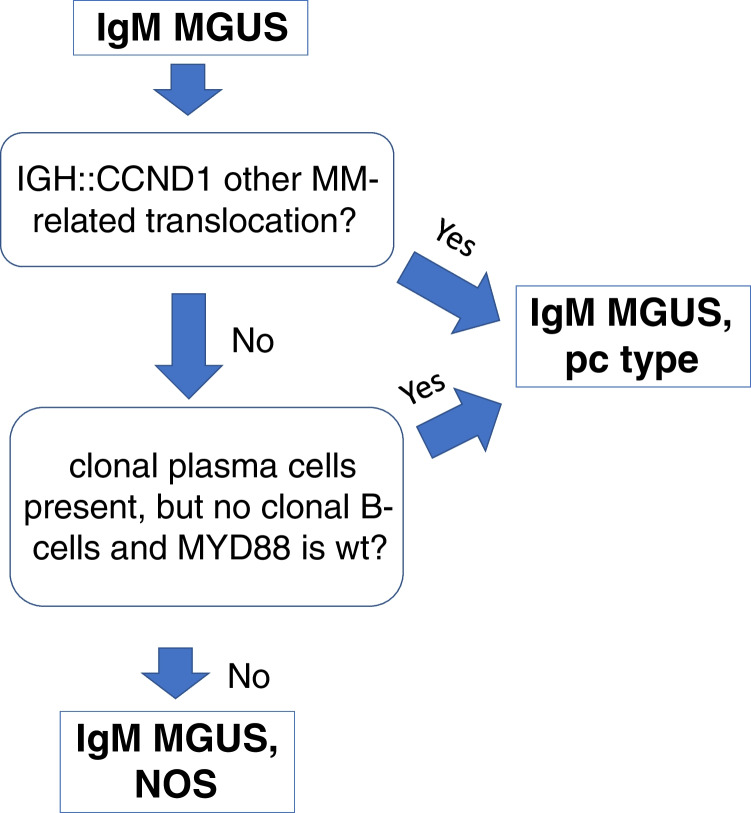
Defining features of IgM MGUS subtypes in the International Consensus Classification

**Fig. 2:**
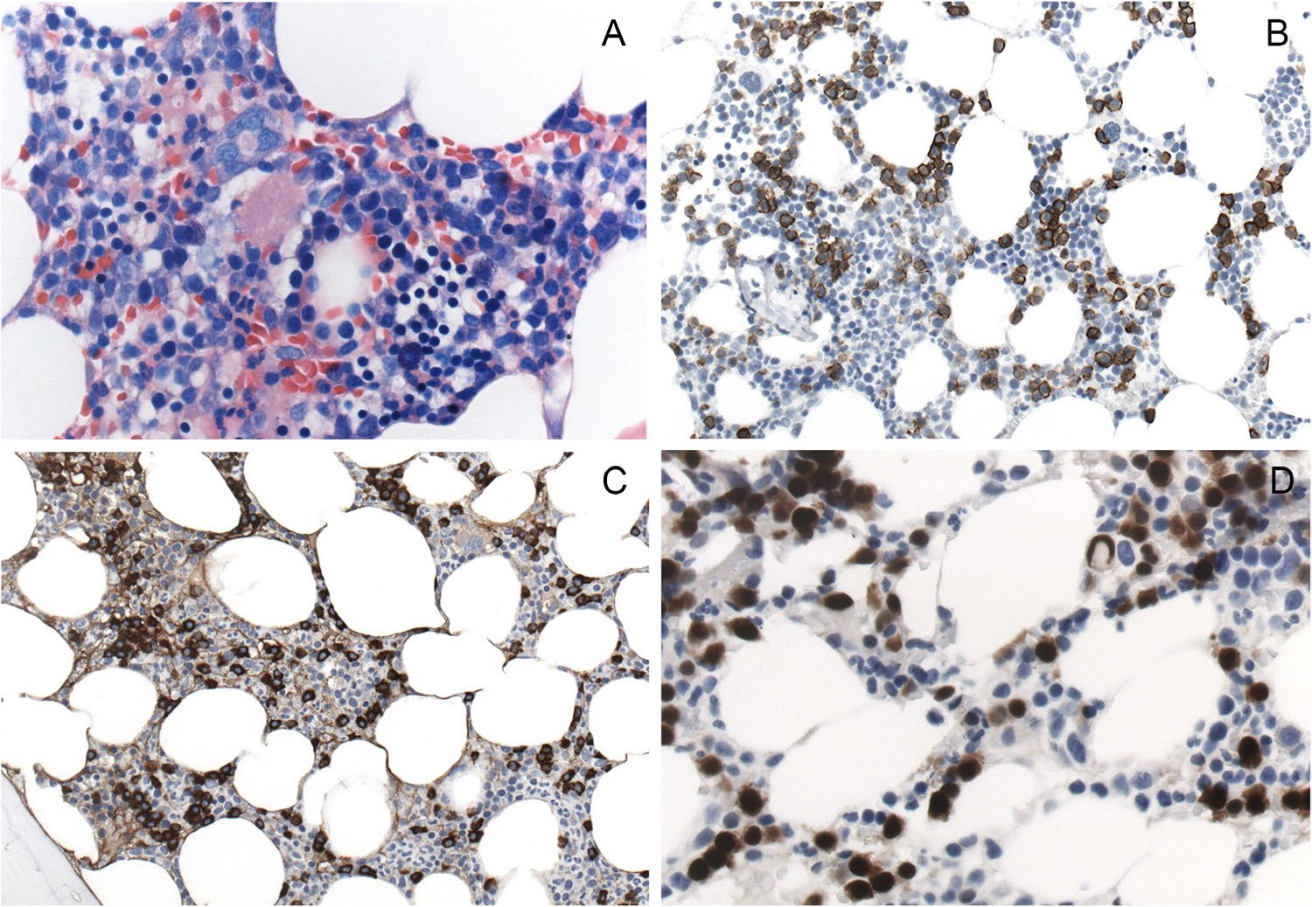
Smoldering myeloma of IgM type. **A** The Giemsa stain shows a subtle, hard-to-identify interstitial infiltrate of plasma cells (original magnification × 400), **B** highlighted in the CD138 stain (× 200), **C** strong expression of IgM (× 200), and **D** strong and homogenous nuclear cyclin D1 (× 400) as evidence for the presence of a t(11;14) translocation, which is present in > 80% of IgM + multiple myeloma. In contrast to many other cases of MM with t(11;14), IgM MM with t(11;14) usually lacks lymphoplasmacytic morphology and expression of B-cell markers. *MYD88* was wild type

The diagnosis of IgM monoclonal gammopathy of undetermined significance (IgM MGUS) is established in cases of IgM paraprotein with < 10% bone marrow clonal plasma cells and lacking lymphoplasmacytic B-cell aggregates sufficient for a diagnosis of LPL. While most cases of IgM MGUS represent potential precursors to LPL or other B-cell neoplasms [35], the rare cases of IgM MM [14, 47] are also presumed to develop through an MGUS precursor stage. In an effort to better identify the rare cases that may progress to multiple myeloma, the ICC [13] recognizes two subtypes of IgM MGUS (Fig. 1). IgM MGUS of plasma cell type is defined as those cases demonstrating t(11;14)(q13;q32) or other cytogenetic abnormalities typical of MM, or as clonal plasma cells without a detectable B-cell component and with wild-type *MYD88* (Fig. 2). All remaining cases should be diagnosed as IgM MGUS, not otherwise specified (NOS). The category of IgM MGUS, NOS therefore includes all cases with a *MYD88* mutation, those with detectable monotypic/monoclonal B-cells but without abnormal lymphoplasmacytic aggregates diagnostic of LPL or other overt small B-cell neoplasm, and cases lacking a detectable plasma cell or lymphoid component. Routine fluorescence in situ hybridization (FISH) studies on enriched plasma cells and *MYD88* mutation analysis are recommended in the evaluation of IgM monoclonal gammopathies in order to distinguish between these two IgM MGUS subsets.

## Lymphoplasmacytic lymphoma

Lymphoplasmacytic lymphoma (LPL) is defined as a neoplasm of small B-cells, plasmacytoid lymphocytes, and plasma cells that does not meet diagnostic criteria for any other recognized small B-cell neoplasm. LPL typically involves the bone marrow, and there may be involvement of lymph nodes, spleen, and peripheral blood, and occasionally extranodal sites. In most cases, LPL is associated with the secretion of a monoclonal IgM paraprotein (i.e., Waldenstrom’s macroglobulinemia (WM)), which is defined as an IgM paraprotein at any concentration associated with LPL involving the bone marrow. A minority of LPL express IgG or IgA rather than IgM, and this finding does not alter the pathologic diagnosis.

Bone marrow aspirate smears characteristically show increased small lymphocytes, plasmacytoid cells, and plasma cells, often associated with increased numbers of mast cells. In trephine biopsies, LPL shows infiltrates of lymphoplasmacytic cells which may show nodular, interstitial, diffuse, or paratrabecular growth patterns. In the 2016 WHO classification [70], abnormal lymphoplasmacytic infiltrates were required to represent at least 10% of the bone marrow to establish a diagnosis of LPL. This requirement stood in contrast to the longstanding International Workshop on Waldenstrom’s Macroglobulinemia definition of LPL/WM [57], which allowed for recognition of LPL involving < 10% of bone marrow cellularity. To resolve these discrepant definitions, the ICC, following extensive discussion, determined that a diagnosis of LPL may be rendered in cases with abnormal lymphoplasmacytic aggregates in the bone marrow and evidence of clonal B-cells and plasma cells, even when the aggregates represent < 10% of cellularity of the trephine biopsy. It is recognized that some cases will remain diagnostically challenging. In such cases, when it is unclear whether lymphoid aggregates are reactive rather than neoplastic, a diagnosis of IgM MGUS may be most appropriate. In lymph nodes, the most classic cases display partial preservation of lymph node architecture with patent sinuses and a diffuse infiltrate of monotonous small lymphocytes with mature chromatin and scant cytoplasm. Plasmacytoid lymphocytes and plasma cells are present, typically representing a minority of the cellularity, and Dutcher bodies are typically present. Less commonly, cases may show more complete architectural effacement, sometimes with vague nodularity or even follicular colonization.

LPL displays a non-specific phenotype that overlaps with that of other entities, especially marginal zone lymphoma. LPL is typically negative for CD5, CD10, and CD23 although CD5 and CD23 may be expressed in a subset of cases. CD25 and CD38 expressions are frequently present. Unlike multiple myeloma, the plasma cells of LPL are typically positive for CD19 and negative for CD56. While studies to date are limited, IRTA1 expression, seen in a subset of cases of marginal zone lymphomas, appears to be rare in LPL [23, 74]. CD180 expression has also been reported to favor a diagnosis of marginal zone lymphoma over LPL [53, 55].

Molecular studies for *MYD88* and *CXCR4* mutations are strongly encouraged in the workup of suspected LPL. *MYD88* mutations in the TIR domain are found in > 90% of LPL predominantly L265P, although rarely non-L265P variants may be present [28, 72]. Molecular studies for *MYD88* L265P should be performed using a sensitive method as some sequencing methods may yield false negative results, especially in cases with low tumor volume [18]. A small percentage of LPL are *MYD88* wild-type with alternative mutations downstream of MYD88 in the NFKB signaling pathway. The CAC debated the question of whether a *MYD88* mutation should be required for the diagnosis of LPL, but the consensus conclusion was that there is insufficient evidence to support the classification of *MYD88* wild-type cases as a distinct entity. Therefore, although neither present in 100% of cases nor not specific for LPL, the presence of *MYD88* L265P mutations assists in the diagnosis of LPL in the appropriate clinicopathologic context, especially as this variant is only seen in a small percentage of marginal zone lymphomas. *CXCR4* mutations are identified in up to 40% of LPL, and, particularly, the non-sense variants have been associated with symptomatic hyperviscosity and resistance to ibrutinib therapy [15, 72]. Of note, the molecular techniques employed should be of sufficient sensitivity to detect *MYD88* and *CXCR4* mutations down to the 1–2% level, such as allele-specific PCR, digital droplet PCR, or highly sensitive NGS-based approaches [31].

## Primary cold agglutinin disease

Primary cold agglutinin disease (pCAD) is recognized as a new diagnostic category, distinct from LPL or IgM MGUS. pCAD is defined as an indolent bone marrow–based lymphoproliferative disorder associated with the production of a cold agglutinin monoclonal antibody [4, 5, 61]. The monoclonal antibody is typical of the IgM isotype with the utilization of the IGHV4-34 variable region and recognition of the I blood group antigen. Notably, cases of CAD associated with nodal involvement by an overt lymphoma should be considered secondary CAD and are excluded from this category. Similarly, the presence of splenomegaly suggests a diagnosis of splenic marginal zone lymphoma or other splenic lymphomas with secondary CAD rather than a diagnosis of pCAD.

Morphologically, bone marrow findings in pCAD are variable. Most cases are reported to show an infiltrate of small lymphocytes in aggregates or nodules with median infiltration of 10% of intermedullary space [61]. A minority of cases may show only scattered B-cells and plasma cells. Prominent plasmacytoid lymphocytes and increased mast cells, typical of LPL, are reported to be absent.

Using immunohistochemistry and flow cytometry, the B-cells are light chain restricted with the expression of CD20, PAX5, CD79b, and FMC7 [61]. A minority of cases are reported to be positive for CD5. The B-cells are negative for CD10, BCL6, and cyclin D1. Light chain–restricted plasma cells are also found. pCAD lacks the *MYD88* L265P mutation found in LPL [49, 50, 61], a feature which greatly facilitates appropriate diagnosis. Cytogenetic studies and chromosomal microarray studies have shown that trisomies of chromosomes 3, 12, and 18 are highly recurrent in pCAD [49]. Next-generation sequencing studies have identified recurrent mutations in *KMT2D* and *CARD11* [50]. These molecular cytogenetic results have suggested that pCAD may be more closely related to marginal zone lymphomas rather than LPL, but there is insufficient evidence of support for the classification of pCAD as a definitive marginal zone lymphoma subtype at this time.

## Plasma cell neoplasms

Plasma cell neoplasms are derived from terminally differentiated B-cells, and plasma cells without a B-cell component constitute the dominant and proliferating cell population, which sets them apart from lymphoplasmacytic lymphoma and other mature B-cell lymphomas, many of which can show maturation to plasma cells to a varying degree [54]. Three groups of disorders can be discerned based on their clinical presentation: (1) multiple myeloma (MM) (previously plasma cell myeloma [58]), the most common disease, characterized by diffuse bone marrow infiltration and diverse manifestations of organ damage; (2) localized plasma cell tumors, namely solitary plasmacytoma of bone (SPB) and primary extraosseous/extramedullary plasmacytoma (EMP), which lack significant bone marrow infiltration; and (3) disorders primarily characterized by the consequences of abnormal immunoglobulin deposition, namely immunoglobulin light chain amyloidosis and non-amyloid light and/or heavy chain immunoglobulin deposition diseases [54]. The presence of a clonal immunoglobulin of non-IgM type, including light chains only, in the absence of diagnostic criteria for one of the above disorders is called non-IgM MGUS, which is considered a virtually universal precursor lesion to MM [35, 75]. Although MGUS is considered a preclinical precursor lesion in asymptomatic individuals, secreted clonal immunoglobulin may cause clinical symptoms and require treatment as described below. Of practical importance, plasma cell neoplasms require the integration of clinical and laboratory parameters, as well as morphology and immunophenotype and cytogenetics to reach a final diagnosis for clinical management. The 2014 IMWG consensus criteria adopted by the revised 2016 WHO classification and the 2022 ICC are summarized in Table 1 [13, 60, 70].

**Table 1:** Classification of plasma cell neoplasms according to ICC 2022 (modified from [60])

	Definition	Progression rate to MM
Non-IgM MGUS	Serum M-protein (non-IgM type) < 30 g/L For light chain MGUS: abnormal FLC ratio (< 0.26 or > 1.65) and urinary M-protein < 500 mg/24 h Clonal bone marrow plasma cells < 10%* Absence of CRAB criteria or amyloidosis that can be attributed to the plasma cell proliferative disorder	1%/year 0.3%/year for light chain MGUS
Smoldering myeloma	Serum M-protein > 30 g/L or urinary M-protein > 500 mg/24 h and/or clonal BM plasma cells 10–60% Absence of myeloma-defining events (SLiM-CRAB) or amyloidosis	10%/year
Multiple myeloma	Clonal BM plasma cells > 10% OR biopsy-proven plasmacytoma AND presence of one or more myeloma-defining events - Hypercalcemia - Renal insufficiency - Bone lesions - Clonal BM plasma cells > 60% - Serum-free light chain ratio > 100 - > 1 focal lesion on MRI	
Solitary bone plasmacytoma	Biopsy-proven solitary lesion of bone Absence of SLiM-CRAB criteria, namely lack of further bony lesions	
	- Without minimal marrow involvement (no clonal plasma cells)	10%/3 years
	- With minimal marrow involvement (< 10% clonal plasma cells)	60%/3 years
Solitary extraosseous plasmacytoma	Biopsy-proven solitary lesion of soft tissues Absence of SLiM-CRAB criteria, namely lack of further bony lesions	
	- Without minimal marrow involvement (no clonal plasma cells)	6%/3 years
	- With minimal marrow involvement (< 10% clonal plasma cells)	20%/3 years
Immunoglobulin light chain amyloidosis (AL)	Presence of an amyloid-related systemic involvement/syndrome (renal, liver, heart, nerve, GI tract, etc.) Positive amyloid staining by Congo red in any tissue Evidence that amyloid is light-chain-related established by direct examination of the amyloid Evidence of a clonal systemic plasma cell proliferative disorder	NA
Localized AL amyloidosis	Localized deposition of AL amyloid in any organ* Absence of criteria for a systemic amyloid-related syndrome Absence of amyloid deposits at other sites (fat pad, bone marrow) Absence of a manifest systemic plasma cell or B-cell proliferative disorder ^#^	Rare (< 2% in 5 years)

*MGUS*: monoclonal gammopathy of undetermined significance. *: May occasionally show multiple sites of amyloid in one organ, e.g. skin. #: a minor M-protein is detected in 0-34% of patients depending on series

## Non-IgM MGUS

The diagnosis of non-IgM MGUS is established in the presence of a minor clonal plasma proliferation in the bone marrow associated with the secretion of a paraprotein of non-IgM type or a single light chain as defined by the International Myeloma Working Group (IMWG) in 2014 and adopted in the ICC 2022 [13, 60]. In brief, the criteria include less than 10% clonal plasma cells in the BM, serum monoclonal protein (non-IgM type) < 30 g/L, or abnormal free light ratio (< 0.26 or > 1.65) and urinary monoclonal protein < 500 mg/24 h in light chain MGUS (approximately 20% of cases), absence of myeloma-defining end-organ damage such as hyper*C*alcaemia, *R*enal insufficiency, *A*nemia, and *B*one lesions (so-called *CRAB* criteria), or amyloidosis that can be attributed to the plasma cell clone. If a clonal bone marrow plasma cell proliferation not fulfilling MM criteria is accompanied by systemic AL amyloid deposition, a diagnosis of immunoglobulin light chain amyloidosis (AL) is rendered. Of note, detection of amyloid in the setting of MGUS or MM does not equal a diagnosis of immunoglobulin light chain amyloidosis (AL) and requires amyloid subtyping, since occasionally other types of amyloid may be found (e.g., ATTR amyloidosis). Non-IgM MGUS is common and affects approximately 3–4% of individuals > 50 years. The average rate of transformation to MM is around 1% per year [35, 36]. As mentioned above, a small subset of IgM MGUS is considered myeloma-type, lacking a B lymphocyte component and *MYD88* mutation. The role of pathology in non-IgM MGUS lies in the quantification of plasma cells in the bone marrow and the demonstration of light chain restriction and aberrant marker expression by immunophenotyping. Of note, cases of non-IgM MGUS with a low load of clonal plasma cells frequently lack a demonstrable light chain restriction by immunohistochemistry or in situ hybridization.

## Paraneoplastic syndromes associated with plasma cell neoplasms

M-protein produced by the neoplastic plasma cells of MGUS may cause a variety of clinical symptoms not fulfilling diagnostic criteria for MM, but nevertheless may require therapeutic intervention. Renal damage due to toxic immunoglobulin light chains is common in plasma cell neoplasms. Light chain cast nephropathy is a MM-defining CRAB criterion, since most cases fulfill the tumor burden requirements for an MM diagnosis. In addition, renal light chain amyloid deposits lead to a diagnosis of immunoglobulin light chain amyloidosis (AL). However, other forms of kidney damage caused by direct or indirect effects of the monoclonal immunoglobulin do not result in a diagnosis of MM or another B-cell neoplasm, if no other criteria are present. Since these patients nevertheless may require treatment, the term monoclonal gammopathy of renal significance (MGRS) has been coined [42]. In most instances, renal damage is due to the specific properties of the secreted immunoglobulin, which may either result in abnormal deposits in the glomeruli or proximal tubules, crystal formation or activation of complement with glomerular inflammation, or thrombotic microangiopathy [41–43]. In addition to renal impairment, a broad range of extrarenal symptoms caused by the clonal immunoglobulin have been described in the setting of early-stage plasma cell or B-cell neoplasia, and an extension of the concept of MGRS has recently been developed to include extrarenal symptoms and complications caused by the clonal immunoglobulin, termed monoclonal gammopathy of clinical significance (MGCS) [24]. In addition to the effects of Ig deposition, disorders caused by autoantibody activity, complement activation, or cytokine secretion of the neoplastic clone are included. Of note, both MGRS and MGCS include disorders with underlying IgM MGUS as well as non-IgM MGUS. Despite their clinical importance, MGRS and MGCS are not considered separate disease entities in the ICC classification [13], which is centered on the malignancy rather than accompanying paraneoplastic syndromes and separates IgM MGUS and non-IgM MGUS on biological and genetic grounds. However, the terms MGRS and MGCS can be added as a clinical descriptor, since the presence of clinically relevant disease caused by the monoclonal immunoglobulin may indicate the use of therapeutic agents aimed at the neoplastic clone, which would otherwise not be justified by the tumor load.

Two specific paraneoplastic syndromes associated with a clonal plasma cell proliferation are POEMS syndrome and TEMPI syndrome. POEMS syndrome is characterized by a clonal plasma cell neoplasm, frequently osteosclerotic multiple myeloma, and, in 11–30% of cases, lymph node changes of multicentric plasma cell variant of Castleman disease. POEMS stands for *p*olyneuropathy, *o*rganomegaly, *e*ndocrinopathy, *m*onoclonal gammopathy, and *s*kin lesions [20]. Other common features include extravascular fluid overload with effusions, papilledema, lung disease, and markedly elevated levels of vascular endothelial growth factor (VEGF). Most patients (90–95%) show an M-protein with lambda light chains [19]. The bone marrow exhibits a lambda-restricted plasma cell population with concomitant increased polytypic plasma cells, lymphoid aggregates rimmed by plasma cells, and megakaryocyte hyperplasia [17]. TEMPI syndrome (*t*elangiectasias, elevated erythropoietin level and *e*rythrocytosis, *m*onoclonal gammopathy, *p*erinephric fluid collections, and *i*ntrapulmonary shunting) is a rare paraneoplastic syndrome associated with MGUS or MM, which may be confused with polycythemia vera due to erythrocytosis caused be extremely elevated erythropoietin levels [71]. The bone marrow in these patients shows significant erythroid hyperplasia without the features of a myeloproliferative neoplasm and a clonal plasma cell population mostly at MGUS levels [63].

A rare, unusual feature of MM is crystal-storing histiocytosis, a reactive hyperplasia of macrophages containing abundant crystallized IG inclusions of kappa light chain type, which may be confused with a storage disorder and is occasionally also observed in other B-cell neoplasms secreting a clonal IG [29, 39].

## Smoldering multiple myeloma (SMM)

SMM is a clinically asymptomatic disease stage, which was traditionally handled with a wait-and-see approach [37]. SMM shows a higher tumor load than MGUS, with serum monoclonal protein (IgG or IgA) ≥ 30 g/L or urinary monoclonal protein ≥ 500 mg/24 h and/or clonal bone marrow plasma cells 10–60%, but lacks features for symptomatic MM including the so-called SLiM-CRAB criteria following the updated IMWG definitions [60]. The so-called SLiM criteria added in the 2014 IMWG consensus moved some cases previously designated SMM into the active MM category including the *s*ixty % cutoff for bone marrow plasma cells, a *li*ght chain ratio of kappa to lambda > 100, and more than 1 focal lesion on *m*agnetic resonance imaging (MRI). Recent years have shown that the progression risk of SMM, overall about 10%/year and thus tenfold higher than for MGUS, varies widely [37]. Whereas about 50% and 30% of SMM patients do not progress to symptomatic disease within 5 and 10 years, respectively, some patients with SMM have a high progression risk and might benefit from early treatment to avoid the complications of manifest MM. The progression risk can be estimated by conventional clinical and laboratory features, but the addition of cytogenetic and molecular features described below allows for a refined stratification of patients with smoldering MM [9–11, 38, 52]. The CAC therefore strongly recommended using established risk models for stratification of SMM to identify patients requiring therapy without yet fulfilling criteria for symptomatic MM [38, 52].

## Multiple myeloma

**Fig. 3:**
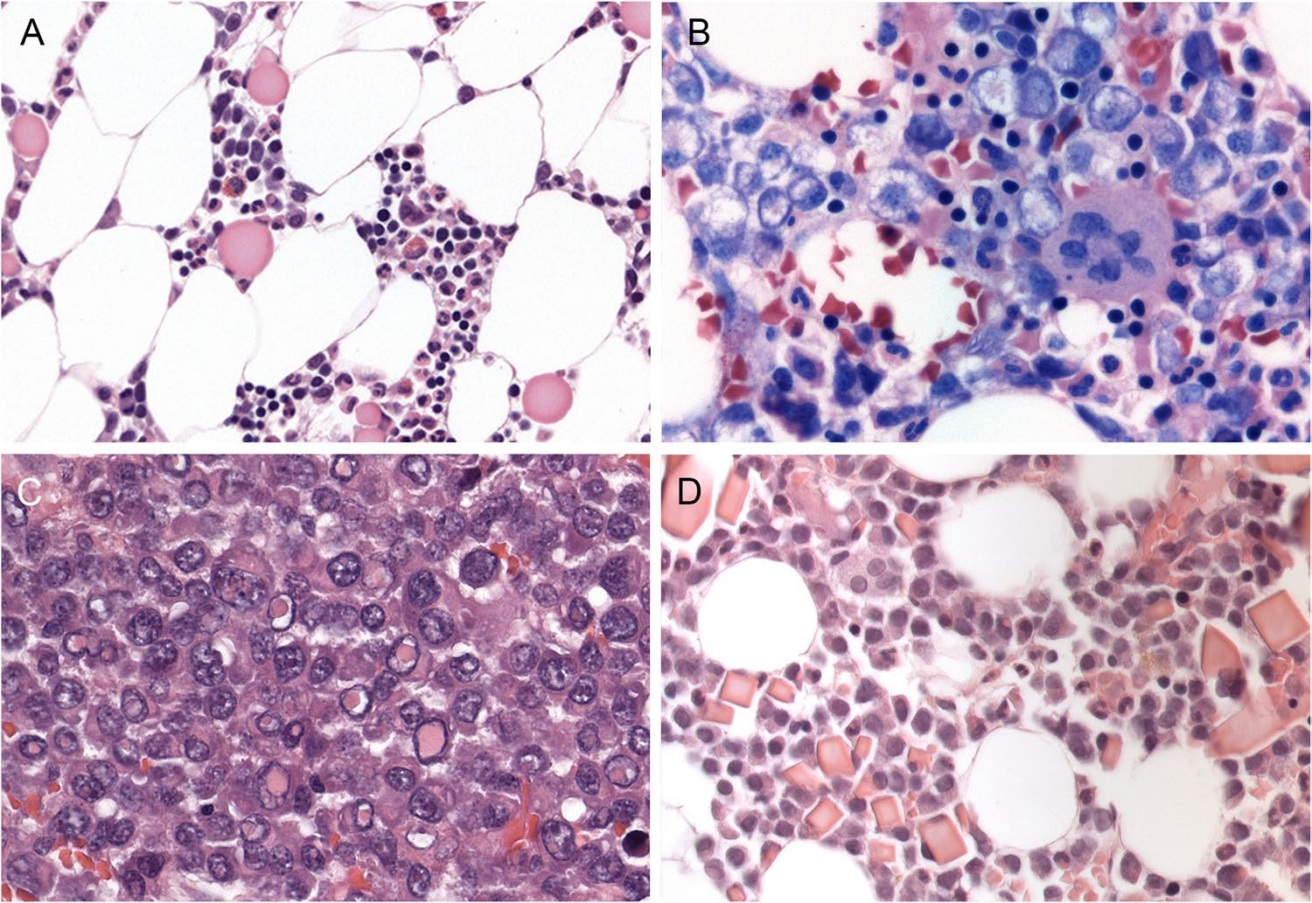
Unusual morphological features in multiple myeloma. **A** Formation of abundant Russell bodies as sign of disturbed IG secretion (original magnification × 200). **B** MM with pseudo-signet ring cells (× 400). **C** MM with significant nuclear pleomorphism and abundant Dutcher bodies (× 400). **D** MM with abundant crystalline IG inclusions (× 200)

Multiple myeloma is one of the most common hematological neoplasms and constitutes about 1% of all human cancers. Manifest or symptomatic MM is diagnosed in patients fulfilling any of the SLiM-CRAB criteria according to the 2014 IMWG consensus as described above and in Table 1. From the diagnostic viewpoint, this means that differentiating between smoldering and manifest MM requires the integration of clinical data, unless the plasma cell infiltrate exceeds 60% of BM cellularity. The plasma cell percentage should be assessed both cytologically on BM aspirates and in the BM trephine, with the higher number taken for diagnosis. Although most cases of MM are easily identifiable on routine stains, cases with lymphoplasmacytic morphology, frequently associated with CD20 expression, anaplastic MM, and cases with unusual cytoplasmic inclusions due to disturbances in IG secretion may cause diagnostic difficulties (Fig. 3). For exact quantification in the BM trephine, it is advised to use immunostaining for plasma cell markers such as CD38, which is also the target for the therapeutic antibody daratumumab and can be downregulated following treatment, CD138 or MUM1, since tumor cell counts especially in cases with small cell or lymphoplasmacytic morphology and interstitial infiltration may be underestimated in routine stains. Of note, CD138 may be weak in some MM cases and is also expressed by a variety of epithelial tumors and may result in misinterpretation, especially in conjunction with CD56 expression. In addition to showing light chain restriction, immunostains and flow cytometric immunophenotyping can demonstrate an aberrant plasma cell phenotype, such as expression of CD56, CD117, or cyclin D1 and loss of CD19. Strongly and homogeneously expressed cyclin D1 can serve as an indicator for the t(11;14) translocation and is associated with lymphoplasmacytic morphology and concomitant expression of CD20 and other B-cell markers usually absent from MM cells in about half the cases, providing a potential diagnostic pitfall [46, 69]. CD56 is expressed in approximately 70–80% of MM, with a lower incidence in t(11;14) + MM and IgM + MM, and can be downregulated in secondary extramedullary spread, especially in plasma cell leukemia.

### Specific subtypes of MM

Some subtypes of MM show specific clinical and/or biological features, which deserve special mention. Most MM secrete a complete or incomplete (light chains (LC) only) monoclonal immunoglobulin (IgG > LC > IgA >  > IgM, IgD, IgE), but a very small subset of non-secretory MM lacks a detectable M-protein in blood or urine, even if very sensitive tests such as the free light chain ratio are used. This phenomenon can be due to either lack of secretion or lack of production of the clonal IG. Patients with non-secretory MM show a distinct clinical profile with the absence of M-protein-induced organ damage, such as renal insufficiency, and lower levels of immunoparesis [22, 45].

IgD + MM is a rare disease subtype (1–2% of MM) derived from preclass switched B-cells and more frequently shows a t(11;14) translocation. Although previously considered to exhibit more aggressive behavior overall, survival is comparable to other MM subtypes with modern therapies [1, 44].

Plasma cell leukemia (PCL), which can arise as primary (60–70%) or less frequently as secondary (30–40%) PCL, is an aggressive form of MM with sometimes acute onset and poor prognosis. According to the recently published consensus of the IMWG, the threshold of circulating plasma cells for a diagnosis of PCL was lowered from ≥ 20 to ≥ 5%, given the similarly poor prognosis of patients fulfilling these new criteria [25].

### Genetic subclassification of MM

MM shows a significant genetic heterogeneity, which has been recognized in the last decades. Two main groups are defined by cytogenetics, namely recurrent IGH translocations with a variety of partners in 40–50% of cases and up to 55% lacking recurrent translocations but exhibiting hyperdiploidy, with infrequent cases not falling into either group [6, 26, 27]. These cytogenetic features are already present in MGUS and thus represent primary alterations [21, 30]. They persist throughout the disease course and are strongly correlated with clinical and phenotypic features, prognosis, therapy response, and the gene expression profile [58, 64, 73, 76]. Therefore, the 2022 ICC proposes to formally subdivide MM into 2 mutually exclusive groups, namely (1) MM NOS and (2) MM with recurrent genetic abnormalities including MM with *CCND* family translocations, MM with *MAF* family translocation, MM with *NSD2* translocation, and MM with hyperdiploidy, characterized by trisomies of uneven chromosomes (3, 5, 7, 9, 11, 15, 19, and 21) (Table 2) [13]. Of note, these genetic groups are also of importance for risk stratification models of MM, which incorporate the high-risk t(4;14) and t(14;16) translocations and also secondary high-risk alterations such as del(17p), amp1q, and del(1p) [58, 68]. The current standard for the detection of these cytogenetic aberrations is interphase fluorescence in situ hybridization (FISH), and minimal and comprehensive FISH panels for the evaluation of MM have been published [16, 58]. At primary diagnosis including smoldering MM, FISH should be performed at least for the t(4;14) and t(14;16) translocations, for alterations of chromosome 1 (+ 1q, -1p) and -17p, usually complemented by the t(11;14) and odd-numbered chromosomes for detection of hyperdiploidy. These alterations may also be detected more comprehensively with gene expression profiling (GEP) or WGS. GEP has also been used to refine prognostication by defining risk scores such as the GEP70 or the EMC92 [34, 67].

**Table 2:** Genetic classification of MM according to ICC 2022 [13]

	Frequency	Prognostic Impact
Multiple myeloma NOS	9–10%	
MM with recurrent genetic abnormality	90%	
- MM with *CCND* family translocation	- 18–20%	Standard risk
- t(11;14) *CCND1::IGH*	- 16%	
- t12;14) *CCND2::IGH*	- < 1%	
- t(8;14) *CCND3::IGH*	- 2–6%	
- MM with *MAF* family translocation	- 6–8%	Poor risk
- t(14;16) *IGH::MAF*	- 3–5%	
- t(8;14) *MAFA::IGH*	- 1%	
- t(14;20) *IGH::MAFB*	- 2%	
- MM with *NSD2* translocation t(4;14)	- 13–15%	Poor risk
- MM with hyperdiploidy	- 45%	Favorable

The role of mutational profiling in MM, which shows a very heterogeneous molecular landscape, remains to be determined in future studies, but mutational analysis of *TP53* is recommended for relapsed MM [40, 51, 59]. Of note, the mutational profile is predetermined by the primary cytogenetic alterations, pointing to an oncogenic interdependence of primary and secondary genetic alterations with non-random clonal evolution [73]. A factor potentially complicating risk prediction and therapy planning is the significant spatial heterogeneity with the development of subclones restricted to focal lesions as demonstrated by profiling of multiple lesions from individual MM patients [62]. Another important feature of the malignant plasma cells in MM is the interaction with and dependence on the bone marrow microenvironment, which gradually diminishes over the course of the illness and finally can result in extramedullary extension of disease and secondary plasma cell leukemia [65].

## Localized plasma cell neoplasms

**Fig. 4:**
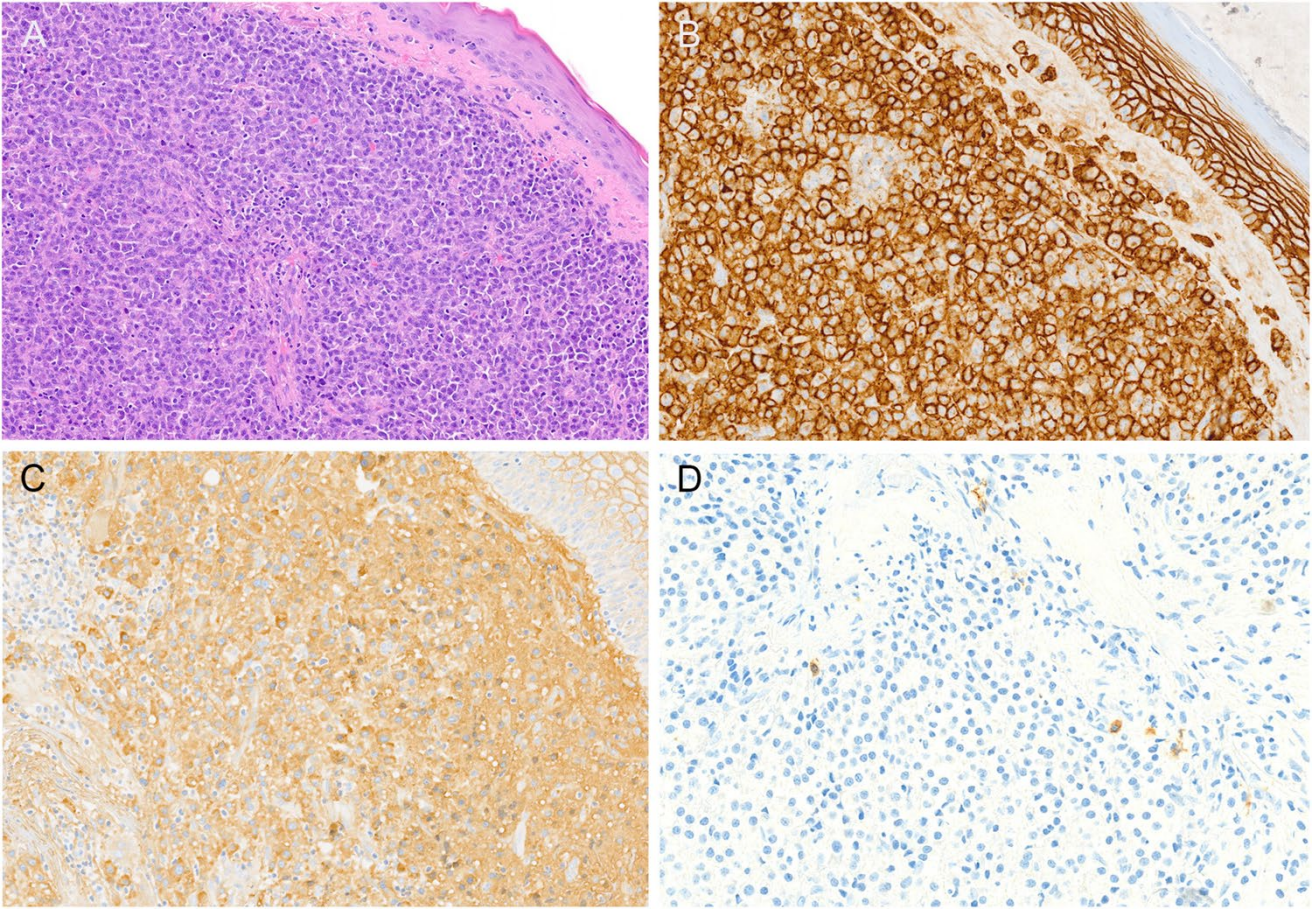
Morphology and immunophenotype of primary extramedullary plasmacytoma of the larynx. **A** Monotonous sheets of well-differentiated plasma cells below squamous epithelium (original magnification × 200x). **B** Strong expression of CD138, which is also positive in the squamous epithelium (× 400). **C** Monotypic expression of kappa light chains (× 400) and **D** rare residual reactive lambda positive plasma cells (× 400)

Two forms of localized plasma cell neoplasms are recognized: solitary plasmacytoma of bone (SBP) and primary extraosseous/extramedullary plasmacytoma (EMP) [70]. Both are characterized by tumor-forming accumulations of clonal plasma cells with the absence of manifest bone marrow infiltration by definition. SBP accounts for about 4–5% of all plasma cell neoplasms and lacks MM-defining criteria or clonal bone marrow plasma cells > 10% [12, 56]. Solitary EMP comprises only 1–3% of plasma cell malignancies and usually presents at mostly extranodal sites, frequently in the head and neck region. A special variant of EMP with IgA expression predominantly manifests in lymph nodes of the head and neck region, frequently in younger patients with various forms of immune dysregulation [66]. Both SBP and EMP are characterized by monotonous proliferations of mostly mature plasma cells with light chain restriction (Fig. 4). For EMP, the presence of an accompanying neoplastic B-cell population raises the differential diagnosis of an extranodal marginal zone lymphoma (Table 3). Since amyloid deposition frequently accompanies EMP, a Congo red stain should be included in the workup. Phenotypically, EMP, SBP, and MM are very similar, but EMP shows less frequent expression of CD56, usually absence of cyclin D1 (and the t(11;14) translocation), and a lower proliferation rate as compared to extramedullary manifestations of multiple myeloma, which usually represent an aggressive terminal stage of disease and may show high MYC expression and *TP53* aberrations [33]. Extramedullary MM and rarely EMP may show plasmablastic cytology with large nuclei with open chromatin and prominent eosinophilic central nucleoli and high nuclear-cytoplasmic ratio, which raises a differential diagnosis of plasmablastic lymphoma. Table 3 summarizes the criteria for the distinction of B-cell neoplasms with plasmacytic/plasmablastic differentiation. Although data are limited, EMP shows MM-type cytogenetics with recurrent IGH translocations and polysomies, emphasizing a close relationship to MM [7, 8]. Progression to MM occurs in approximately 15% of EMP cases [2, 8, 12, 33, 56]. The risk for progression to MM overall is higher for SBP (60–85% after 10 years) than for EMP (12–35% after 10 years), but it has been shown that the presence of a minimal infiltrate of clonal plasma cells in the BM detected by flow cytometry is associated with a strongly increased progression risk, although the risk remains lower for EMP. The 3-year progression rate to MM is about 60% for SBP with the presence of clonal BM plasma cells versus 6–12% without, and approximately 20% for EMP with and 6% without BM plasma cells. The CAC therefore strongly recommended including the absence or presence of minimal BM infiltration into the diagnosis and using flow cytometry for staging in every localized plasma cell neoplasm. Due to the rarity of these disorders, the prognostic impact of cytogenetic or other molecular alterations is currently unknown.

**Table 3:** Differential diagnosis of clonal extramedullary infiltrates with plasmacytic/plasmablastic differentiation

	LPL	EMZL	EMP	EMM	PBL
Clinical features	Systemic disease with BM infiltration and IgM paraprotein in most cases, IgM MGUS NOS as precursor Indolent clinical course, symptoms due to macroglobulinemia	EMZL: frequently localized disease at typical extranodal locations BM involvement rare, excellent prognosis	Localized disease, frequently upper respiratory tract No/minimal BM involvement Excellent prognosis, progression to MM rare (mainly in cases with minimal BM involvement)	Usually in advanced/terminal stage MM, occasionally isolated extramedullary relapse after treatment Poor prognosis	Aggressive disease in HIV infection, iatrogenic IS, immunocompetent elderly Frequently extranodal (oral cavity, GI tract), 50% nodal in immunocompetent patients
Cytology	Lymphocytes and lymphoplasmacytic cells, variable amounts of PCs	Small lymphs, centrocytoid or monocytoid cells, variable amounts of PCs	Mostly mature PCs in monotonous sheets	Sheets of PCs, anaplastic or plasmablastic features can be observed	Monomorphic proliferation of large cells with prominent nucleoli, usually lack of mature PCs
Phenotype	CD20 + , CD5-/+ , CD23-/+ PC lack CD56, cyclin D1, and CD117 EBV neg	CD20 + , CD5-/+ , CD43-/+ PC lack CD56, cyclin D1, and CD117 EBV neg	PC markers MUM1 + , CD38 + , CD138+ CD79a ± , LC restriction; CD56-/+ (weak), lack of CD20, PAX5, cyclin D1, MYC or p53 overexpression EBV + in up to 15%	PC markers MUM1 +, CD38 + , CD138 + , LC restriction; CD56 ± ; MYC and/or p53 overexpression common, high MIB-1 EBV usually neg	PC markers MUM1 +, CD38 + , CD138 +, LC restriction; CD56± ; MYC and/or p53 overexpression common, high MIB-1 EBV + in 50–75%
Genetics	*MYD88* L265P in > 90%, *CXCR4*^*mut*^ in 30–40% Lack of MM-type translocations	EMZL-type translocations depending on location *TNFAIP3* mutations Lack of MM-type translocations	IGH translocations with exception of t(11;14), MM-type trisomies Mutational spectrum unknown	Primary genetic alterations of MM, enrichment of high-risk cytogenetics, and secondary alterations including del(17p), 1q gains, *MYC* translocations and *TP53* mutations	*MYC* translocation in 50%, lack of MM-type translocations Mutations in RAS-RAF, JAK-STAT, and NOTCH pathways

*LPL*, lymphoplasmacytic lymphoma; *EMZL*, extranodal marginal zone B-cell lymphoma; *EMP*, extramedullary plasmacytoma; *EMM*, extramedullary multiple myeloma; *PBL*, plasmablastic lymphoma; *BM*, bone marrow; *IS*, immunosuppression; *EBV*, Epstein-Barr virus; PC, plasma cell

## Immunoglobulin light chain (AL) amyloidosis

**Fig. 5:**
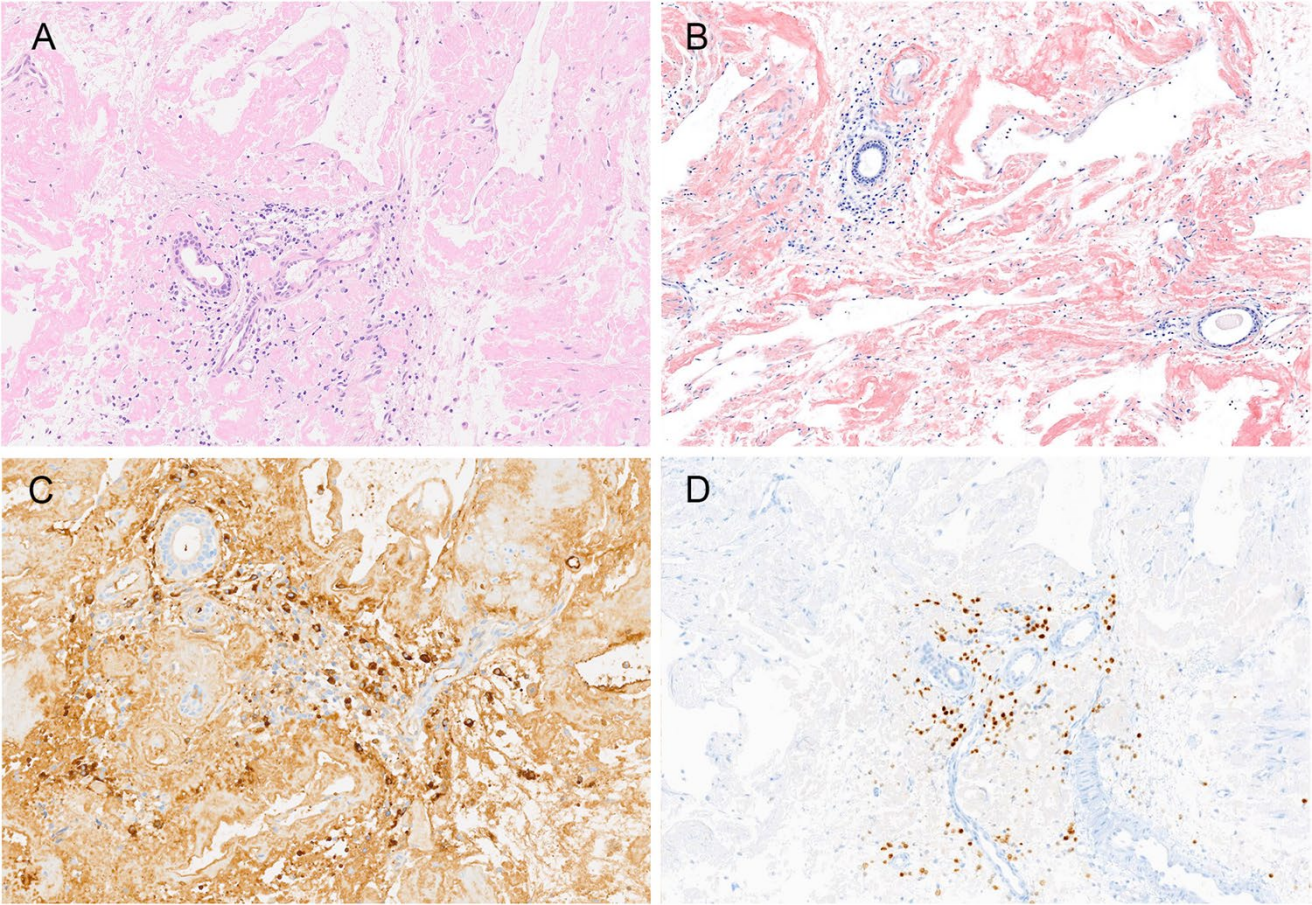
Localized AL amyloidosis of the lung. **A** Amorphous eosinophilic deposits with rare lymphocytes and plasma cells (HE, original magnification × 200). **B** Strong Congo red positivity (× 200). **C** Rare lambda positive plasma cells (× 200) with **D** nuclear MUM1 expression (× 200)

The CAC recommended changing the name of primary amyloidosis to immunoglobulin light chain (AL) amyloidosis to clearly separate it from other forms of amyloid deposition disease, as well as from non-amyloid-forming deposition of clonal immunoglobulin in the light chain and heavy chain deposition diseases, but the diagnostic criteria otherwise remain unchanged (Table 1) [13]. A rare, clinically mostly indolent plasma cell disorder is localized light chain (AL) amyloidosis. Localized AL amyloidosis, also called amyloid tumor or amyloidoma, is characterized by tumor-forming deposits of AL amyloid (lambda light chains in 50–75%) with frequent foreign body-type giant cell reaction in the absence of systemic disease or a manifest, i.e., tumor-forming accompanying B-cell or plasma cell neoplasm (Table 1) [3, 32, 48]. Localized AL amyloidosis may be accompanied by a sparse infiltrate of B-cells and plasma cells with clonality detected by immunohistochemistry or in situ hybridization for IG light chains (Fig. 5), or by PCR for clonal IG rearrangements in about 30% and a small serum M-protein in about 20% of cases, depending on case selection criteria. The most commonly affected organs are the urinary tract, lung, upper respiratory tract, skin, and the GI tract, but virtually any organ can be affected. Local progression is common, but several large retrospective series have demonstrated that progression to systemic IG light chain amyloidosis is very rare (< 2%), and complications, if any, usually arise from local tumor growth potentially requiring surgical intervention [3, 32, 48]. The CAC therefore recommended recognizing localized light chain amyloidosis as a distinct entity in order to separate it from systemic immunoglobulin light chain (AL) amyloidosis, which carries a much graver prognosis and requires systemic therapy [13].
